# Ultrasound-Assisted Extraction Optimization of Phenolic Compounds from *Citrus latifolia* Waste for Chitosan Bioactive Nanoparticles Development

**DOI:** 10.3390/molecules24193541

**Published:** 2019-09-30

**Authors:** Nelly Medina-Torres, Hugo Espinosa-Andrews, Stéphane Trombotto, Teresa Ayora-Talavera, Jesús Patrón-Vázquez, Tania González-Flores, Ángeles Sánchez-Contreras, Juan C. Cuevas-Bernardino, Neith Pacheco

**Affiliations:** 1 Centro de Investigación y Asistencia en Tecnología y Diseño del Estado de Jalisco CIATEJ, A.C. SubsedeSureste, Parque Científico Tecnológico de Yucatán, Km 5.5 Carretera Sierra Papacal-Chuburná Puerto, Mérida CP 97302, Mexico; arapachecol@gmail.com (N.M.-T.); tayora@ciatej.mx (T.A.-T.); qjpatron@gmail.com (J.P.-V.); tgonzalez@ciatej.mx (T.G.-F.); msanchez@ciatej.mx (Á.S.-C.); 2 CIATEJ Subsede Zapopan, Camino Arenero 1227, El Bajío del Arenal, Zapopan Jalisco 45019, Mexico; hespinosa@ciatej.mx; 3 Ingénierie des Matériaux Polymères (IMP), CNRS UMR 5223, Université Claude Bernard Lyon 1, Univ Lyon, F-69622 Villeurbanne, France; stephane.trombotto@univ-lyon1.fr; 4 CONACYT–Centro de Investigación y Asistencia en Tecnología y Diseño del Estado de Jalisco, Subsede Sureste, Parque Científico Tecnológico de Yucatán, Km 5.5. Carretera Sierra Papacal - Chuburná Puerto, Mérida, Yucatán 97302, Mexico; jcuevas@ciatej.mx

**Keywords:** Persian lemon waste, value-added products, Ultrasound-assisted extraction, phenolic compounds, chitosan bioactive nanoparticles

## Abstract

Bioactive Phenols-loaded chitosan nanoparticles (PL-CNps) were developed by ionic gelation from Persian lemon (*Citrus latifolia*) waste (PLW) and chitosan nanoparticles. Response Surface Methodology (RSM) was used to determine the optimal Ultrasound-Assisted Extraction (UAE) conditions for the total phenolic compounds (TPC) recovery from PLW (58.13 mg GAE/g dw), evaluating the ethanol concentration, extraction time, amplitude, and solid/liquid ratio. Eight compounds expressed as mg/g dry weight (dw) were identified by ultra-performance liquid chromatography coupled photo diode array (UPLC-PDA) analysis: eriocitrin (20.71 ± 0.09), diosmin (18.59 ± 0.13), hesperidin (7.30 ± 0.04), sinapic acid (3.67 ± 0.04), catechin (2.92 ± 0.05), coumaric acid (2.86 ± 0.01), neohesperidin (1.63 ± 0.00), and naringenin (0.44 ± 0.00). The PL-CNps presented size of 232.7 nm, polydispersity index of 0.182, Z potential of −3.8 mV, and encapsulation efficiency of 81.16%. The results indicated that a synergic effect between phenolic compounds from PLW and chitosan nanoparticles was observed in antioxidant and antibacterial activity, according to Limpel’s equation. Such results indicate that PLW in such bioprocesses shows excellent potential as substrates for the production of value-added compounds with a special application for the food industry.

## 1. Introduction

The disposal of food wastes leads to environmental and economic concerns, due to its high fermentability, high transportation costs, lack of disposal sites, and the difficulties to accumulate organic wastes for a long time [1]. Industrialization of citrus fruits, particularly lemon, creates large quantities of by-products, which can be of interest to the food industry due to containing high levels of vitamin C and phenolic compounds such as eriocitrin, hesperidin, neohesperidin, naringin, and diosmin [2]. The biological potential of phenolic compounds lies in their antioxidant activity, associated to the ability of the compounds to reduce reactive oxygen species, and the antimicrobial activity related to inhibition of cytoplasmic bacterial membrane function [3,4].

For the recovery of phenolic compounds, conventional techniques including maceration and Soxhlet extraction have been used; however, they presented disadvantages that limit the application of the extracts due to solvent toxicity [5]. The implementation of innovative extraction technologies such as UAE has been promoted, the principle of which is the acoustic cavitation improving the extraction yield and kinetics with a significant reduction in temperature, solvent consumption, and extraction time [6,7]. During ultrasound-assisted extraction (UAE), several variables must be considered such as time, temperature, type, and concentration of the solvent. The simultaneous study of these variables can be performed through the response surface methodology (RSM), a mathematical and statistical tool used to determine the optimal UAE conditions for the recovery of phenolic compounds from various sources [8]. 

On the other hand, it is well known that the biological activity of phenolic compounds can be affected by light, high temperatures (>50 °C), and sudden changes in pH. Consequently, several systems have been developed to protect the phenolic biological activity such as encapsulation, which is a system where the compounds can also be released at controlled rates. Nanoparticles (Nps), characterized by a smaller size from 1 nanometer to 1 micrometer obtained by encapsulation, have been used as a protection mechanism to improve the solubility, absorption, and bioavailability of phenolic compounds, and decrease the risk of their degradation [9]. Ionic gelation technology is an encapsulation method based on the solid-gel transition of biopolymer in the presence of polyanions with a high density of negative charges such as citrate, sulfate, and phosphate ions. The Nps based on chitosan (CNps) by ionic gelation has proved to be an excellent alternative for the encapsulation of different phenolic compounds such as chlorogenic acid [10], gallic acid [11], and ferulic acid [12], with encapsulation efficiency values of 14.71–85%. These authors have suggested that encapsulation efficiency is greatly influenced by molecular weight and chitosan concentration as well as the different polarities of the phenolic compounds.

Hence, the main objective of this work was to: a) evaluate UAE conditions including the ethanol concentration, extraction time, amplitude, and solid-liquid ratio on total phenolic compounds (TPC) recovery from Persian lemon waste (PLW) using RSM, b) identify and quantify the phenolic compounds present in the PLW by using ultra-performance liquid chromatography coupled photo diode array (UPLC-PDA) analysis, and c) structural characterization of Phenol-loaded chitosan nanoparticles (PL-CNps) developed by ionic gelation and evaluate its biological activity. 

## 2. Results and Discussion 

### 2.1. Modeling and Optimization of UAE for TPC Recovery 

Experimental and predicted values obtained from the analysis of the randomized Box–Behnken design to evaluate the effect of the main variables: ethanol concentration (X_1_), extraction time (X_2_), amplitude (X_3_), and the solid/liquid ratio (X_4_) for TPC recovery from PLW through the UAE process, are presented in Table 1. TPC recovery from experimental values ranged from 8.10 ± 0.01 to 17.84 ± 0.01 mg GAE/g dw. According to the Box–Behnken design, the highest TPC recovery was obtained at ethanol concentration (50%), extraction time (12.5 min), amplitude (80%) and solid/liquid ratio (1g/50 mL). The ANOVA analysis showed an R^2^ value of 0.930, which was statistically acceptable at a 95% confidence level. The results indicated that the independent variables X_1_, X_3_, and X_4_ had linear effects (*p* < 0.05), X_2_ and X_3_ showed a quadratic effect (*p* < 0.05), while interaction effects (*p* < 0.05) were observed for A) X_1_ X_3_, B) X_1_ X_4_, and C) X_2_ X_3_. Regression coefficients from significant effects (linear, quadratic, and interaction) were obtained by the multiple regression analysis. The second-order polynomial equation, Equation (1) was formulated with the significant regression coefficients at 95% of confidence level (*p* < 0.05):

(1) $$\mathrm{TPC}\;(y)\;=\;-114.493\;+\;0.325\;X1_{\;}+\;5.878\;X_{2}\;+\;1.214\;X_{3}\;+\;1.187\;X_{4}\;+\;0.004\;X_{1}X_{3}\;-0.015X_{1}X_{4}-0.108{X_{2}}^{2}-0.040X_{2}X_{3}-0.005\;{X_{3}}^{2}.$$

The maximum predictive value of TPC recovery and the optimum condition of each variable were determined according to a second-order polynomial equation. The optimal conditions obtained were ethanol concentration (50%), extraction time (10.40 min), amplitude (89.04%) and solid/liquid ratio (1 g/50 mL). Under these conditions, the optimal predicted TPC value was calculated as 17.30 mg GAE/g dw, which was within the range of TPC (10.55–19.90 mg GAE /g dw) reported by Ubando-Rivera et al. [13] on Mexican lemon peel (*C. aurantifolia*). 

### 2.2. Analysis of Extraction Variables of UAE 

#### 2.2.1. Individual Effects of Ethanol Concentration, Extraction Time, Amplitude, and the Solid/Liquid Ratio

The results obtained showed that the optimum ethanol concentration was 50%. This result was similar to the better conditions reported by Ledesma-Escobar et al. [14] for TPC recovery from *Citrus latifolia* fruit evaluated at different growth stages and extracted by UAE. Hydroalcoholic solvents are considered a suitable system for extraction, due to the different polarities of the phenolic compounds [8]. Drosou et al. [15] reported that a support mixture of ethanol-water allows a synergic effect between the solvents. These authors indicated that water acts as a swelling agent of the plant matrix increasing the contact surface, while the lower ethanol polarity favors the solubility and diffusion of the phenolic compounds by reducing the dielectric constant of the solvent, thereby inducing the rupture of the bond between the solutes and the matrix. The optimal UAE time was obtained at 10.40 min of sonication. This result is similar to that reported by Sahin et al. [16], who worked with phenolic extracts of olive leaves which indicated that the extraction process can reach up to 90% of recovery during the first 10–20 min of extraction, calling this period a “wash” stage where the dissolution of the soluble components from the surface of the matrix happens. Although the same authors mention that there is a second stage known as slow extraction (which lasts for up to 60 min), it must be considered that during the process it is important to minimize the extraction time to reduce the energy costs. The results indicated an increase in TPC in line with the increase in amplitude until 89.04%. This may be attributed to the intensification of extraction due to the increment in the number of compression and rarefaction cycles of the ultrasonic waves at higher amplitudes, which produce a higher delivery of compounds [8]. Carrera et al. [17], who indicated significant differences of TPC recovery, have previously reported this behavior when comparing amplitudes of 20% and 100%, also suggesting that a greater ultrasonic amplitude improves the extraction process. The optimum value of the solid/liquid ratio (1 g/50 mL) was similar to the reported by Sousa et al. [18] during UAE of phenolic compounds from *Phyllanthus amarus*, who indicated that a ratio of 1 g/40 mL was suitable to allow the penetration of the solvent to the cells, improving the permeation of phenolic compounds. The above indicates that the solid/liquid ratio is one of the most critical factors during mass transfer, since the solvent helps to accelerate the diffusion process. 

#### 2.2.2. Analysis of Interaction Effects 

**Figure 1:**
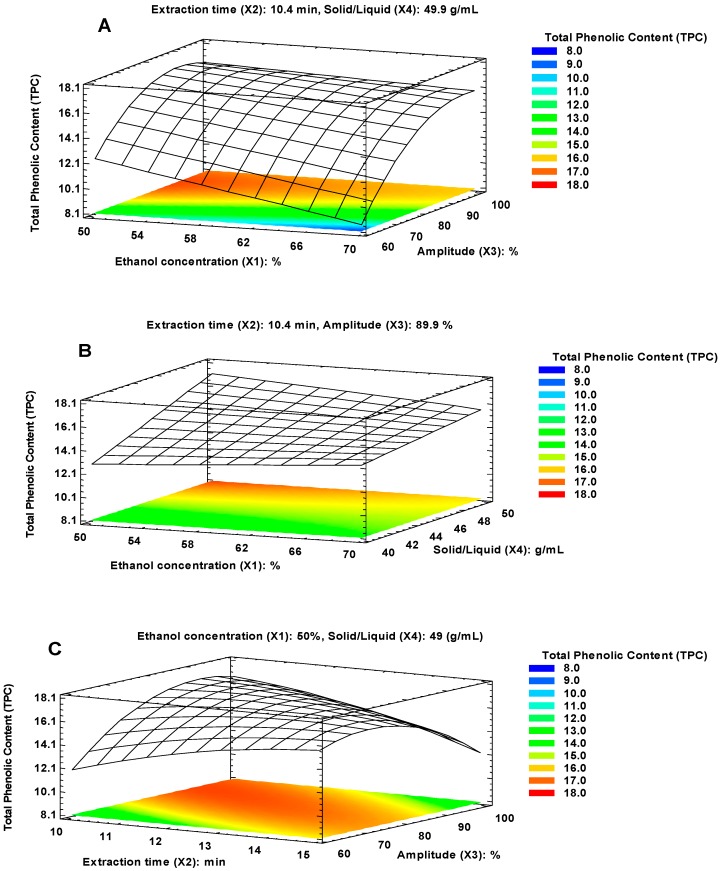
Response surface plots showing the interaction effects over TPC recovery by varying two factors, keeping the other two factors at a constant point. (**A**) Ethanol Concentration-Amplitude (X_1_X_3_); (**B**) Ethanol concentration-solid/liquid ratio (X_1_X_4_); (**C**) Extraction Time-Amplitude (X_2_X_3_).

Figure 1 (A, B, and C) shows the surface response plots, which describes linear, quadratic and interactive effects of independent variables on TPC. The results indicated that the ethanol concentration (X_1_) and the solid/liquid ratio (X_4_) presented linear effects while Sonication time (X_2_) and amplitude (X_3_) showed quadratic effects. Figure 1A shows the interaction of a linear effect (X_1_: ethanol concentration) and a quadratic effect (X_3_: amplitude), where the maximum value of TPC by UAE is obtained at X1: ethanol 50% and X_3_: amplitude 89.04%. The interaction of these parameters resulted in a higher TPC recovery when lower ethanol concentrations is obtained and higher amplitude values were used [8]. On the other hand, Figure 1B shows the interaction of two linear effects (X_1_: ethanol concentration and X_4_: solid/liquid ratio). This interaction indicated an increased in TPC as the solid/liquid ratio and ethanol concentration decreased, similar behavior was presented by Sahin and Samli, who presented a mayor extraction yield in olive leaves when decreasing the solvent concentration and reducing the solid/liquid ratio. This may be influenced by the polarity of the solvent at a lower concentration that favors extraction and a major quantity of solvent when solid/liquid ration decreases [16]. Finally, Figure 1C describes the interaction of two quadratic effects (X_2_: extraction time and X_3_: amplitude), where the curvature of both variables can be observed. The X_2_X_3_ interaction indicates that the highest TPC is obtained above the lower level (X_2_:10 min and X_3_: 60%) but below the upper level (X_2_:15 min and X_3_: 100 %) of each factor. This is also similar to the reported by Sahin and Samli, where extraction time presented a mayor TPC recovery with all the factors evaluated when higher values were tested [16].

### 2.3. Phenolic Compounds Identification and Quantification by UPLC-PDA Analysis 

The chromatographic phenolic profiles of the PLW extracts obtained by maceration and UAE are presented and compared in Table 2. The UPLC-PDA analysis at a wavelength of 290 nm indicated the presence of eight phenolic compounds in both extracts: catechin, eriocitrin, *p*-coumaric acid, sinapic acid, diosmin, hesperidin, neohesperidin, and naringin, which were confirmed and quantified through analytical standard curves. The major compounds for both extracts were hesperidin and eriocitrin. These compounds have been previously reported in another Mexican lemon (*Citrus aurantiifolia*), where the content of hesperidin and eriocitrin was 15.64 and 1.38 mg/100 g dw, respectively [19]. 

The results indicated an improvement of 80% on the recovery of TPC using UAE (58.13 ± 0.38 mg/g dw) to compare maceration extraction (11.19 ± 0.14 mg/g dw). The higher recovery by UAE was observed for catechin and diosmin, reporting an improvement of 93% and 89%, respectively. These results are higher than those reported by Garcia-Castello [20], who obtained an improvement in UAE of around 40% as compared to conventional extraction for the recovery of flavonoids from grapefruit (*Citrus paradisi L.*) residues. A better efficiency of extraction in UAE may be due to the different mechanisms involved during the process, principally: a) fragmentation, attributed to the collisions between particles and ultrasonic waves, and b) erosion, which improves the accessibility of the solvent by the implosion of the bubbles on the surface of the vegetable matrix. Among other mechanisms reported, sono-capillary, sonoporation, and shear stress are capable of improving the penetration of liquid through the channels and altering the permeability of cell membranes, respectively [8]. 

### 2.4. Encapsulation Efficiency of Phenolic Compounds 

The encapsulation of the phenolic extract obtained by optimal UAE conditions was performed using chitosan and the cross-linking agent TPP, the last one, had the function to create inter and intra-molecular reactions with amino groups positively loaded with chitosan [9]. The encapsulation efficiency (EE) of TPC from PLW was 81.16%, which suggests that the –OH groups from phenolic compounds reacted with the positive amino groups of chitosan. This result was higher than that reported by Pulicharla et al. [21], who performed the encapsulation of phenolic compounds from strawberry using CNps, reporting an EE of 42.35–58.48%.

The individual phenolic compounds in PLW extracts reported differences in %EE. The highest efficiency was obtained by naringin (100%), followed by catechin (96.90%), coumaric acid (88.27%), diosmin (86.02%), sinapic acid (84.50%), eriocitrin (83.41%), hesperidin (67.37%), and neohesperidin (62.51%). These values were similar to that reported by different authors using CNPs such as: chlorogenic acid (%EE: 48–59%) [10], gallic acid (59–85%) [11], and ferulic acid (14.71–56.45%) [12]. The above can be attributed to the different polarities of the phenolic compounds, which influences the associative and repulsive forces between the phenolic compounds and the chitosan chains [22]. Helal et al. [23] point out that in addition to polarity, the molecular weight of phenolic compounds can also influence the retention process in a polymeric matrix through the distribution of polyphenols due to the different shapes of the molecules. 

### 2.5. Phenol-loaded Chitosan Nanoparticles Characterization 

#### 2.5.1. Size Particle, Z Potential, and Polydispersity Index 

The average size particle of the PL-CNps was 232.7±10.7 nm. Zhang and Zhao [24] point out that PL-CNps may be formed due to the decrease in the density of electrons of chitosan (positive charge) after the addition of the phenolic compounds extract (negative charge). Then, an increase in the electron delocalization is produced, minimizing the aggregation of the particles decreasing the size of the complex. This characteristic makes this PL-CNps an excellent alternative for use in the food or pharmaceutical industry since it could present a greater muco-adhesiveness in the intestine due to its smaller size [9]. 

The average value of the Z potential of the PL-CNps was −3.8 ± 0.28 mV, which can be explained due to the neutralization of the positive charge density of the protonated amino groups of CNps by the contribution of negative charge density produced by the phenolic extract (−15.40 ± 0.62 mV). In addition, the results of the PDI (0.182 ± 0.06) suggests that PL-CNps presented very homogeneous size distribution according to Zhang and Zhao [24]. 

#### 2.5.2. Fourier Transformed-Infrared (FT-IR) Analysis 

**Figure 2:**
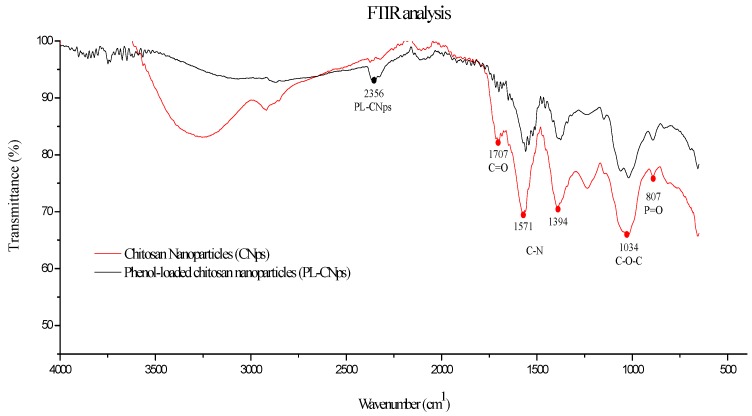
Fourier Transformed-Infrared (FT-IR) spectrum of chitosan nanoparticles (CNps) and Phenol-loaded chitosan nanoparticles (PL-CNps). Obtained using an Agilent Cary 630 spectrometer, in attenuated total reflectance mode.

The FT-IR analysis showed the interactions between CNps and phenolic compounds due to the changes in specific spectral bands. As observed in Figure 2, the broad band was located at 3290 cm^−1^ which was observed for chitosan corresponds with vibration modes of –OH groups [25]. 

On the other hand, the main bands associated with chitosan molecule on CNps were observed at 1571 and 1394 cm^−1^, which was attributed to the stretching of the C–N bonds from amide II and III, respectively. Likewise, the band present at 1034 cm^−1^ may be indicative of the stretching of the C–O–C bond from the chitosan molecule [26]. The band observed at 807 cm^−1^ can be associated to the presence of phosphate groups (P–O, P=O), which could be evidence of the electrostatic interaction between the ammonium ions of chitosan and the phosphorus ions of the TPP [27]. Observations during comparison of spectra from CNps and PL-CNps included: the appearance of a band at 2356 cm^−1^, the disappearance of the band at 1707 cm^−1^ associated to the stretching of C=O from the amide of the chitosan, and a strong deformation of the wideband between 3000 to 3500 cm^−1^ in the PL-CNps. These differences can be attributed to the interaction between the phenolic compounds with the CNps, as indicated by Woranuch and Yoksan [28] in eugenol encapsulation. 

### 2.6. Phenol-loaded Chitosan Nanoparticles Biological Activity

The evaluation of biological activity (antioxidant and antibacterial) of PL-CNps compared to the free PLW extract and CNps as μmTE/g dw and inhibition % is presented in Table 3. The values of antioxidant capacity detected on free PLW extract were similar to the values reported by Esparza-Martínez et al. [29] in lemon wastes (540–839 and 122–683 μm Trolox/g dw for 2,2’-azino-bis(3-ethylbenzothiazoline-6-sulphonic acid) (ABTS) and 2,2-diphenyl-1-picrylhydrazyl radical (DPPH), respectively). The antioxidant activity of phenolic compounds, especially flavonoids, is strongly influenced by their chemical structure, mainly due to the presence of three functional groups: ortho-dihydroxy (catechol) structure in the B-ring; the 2,3-double bond, in conjugation with a 4-oxo function; and the presence of both 3-(a)-and 5-(b)-hydroxyl groups. These groups confer greater stability to the aroxyl radicals by dislocating the electron from the B ring [3]. The CNps showed a low ABTS and DPPH radical scavenging activity (35.18 and < 10 μm Trolox/g dw, respectively), which was similar to that reported by Chen et al. [30]. Although the limited antioxidant properties of CNps have been reported, is important to consider that the antioxidant activity is associated with the chitosan’s molecular weight and degree of deacetylation. On the other hand, PL-CNps showed an increment in antioxidant activity compared to free PLW extract (Table 3).

To determine the importance of the increment on the antioxidant activity and to evaluate the presence of a synergic effect, the expected additive (Ee) response by ABTS and DPPH tests of PLW extract and CNps were calculated using the Limpel’s Equation (Equation (6)). According to the results, the Ee for the ABTS test (57.4 + 9.74 − [(57.4 × 9.74)/100] = 61.55%) was lower than the experimental value of inhibition found by the same test (80.40%). Similar behavior was observed in the DPPH test where Ee (51.8 + 2.23 − [(51.8 × 2.23)/100] = 52.88 %) was lower than the experimental value of inhibition found by the DPPH test (66.0%). Therefore, the combination of PLW extract and CNps presented a synergic effect [31]. Likewise, the combination of PLW extract and CNps presented a better activity antioxidant that the presented by Eshghi and Hashemi [32], for nanochitosan-based coating with copper loaded on physicochemical and bioactive components of fresh strawberry fruit (35% DPPH percentage of inhibition).

CNps efficiency to preserve the antioxidant activity of phenolic compounds has been previously demonstrated in the encapsulation of chlorogenic acid [10], and eugenol [28]. Zhang and Zhao [24] explained that an increase in antioxidant activity can be attributed to changes in the distribution of electrons from phenolic compounds, activating their aromatic hydroxyl groups. 

The antibacterial activity for free PLW extract reported MIC values in the range of 21.17–28.22 μg/mL and presented better activity against Gram-negative bacteria (*Escherichia coli* and *Staphylococcus aureus*). The antimicrobial activity from citrus fruit peels has been attributed mainly to the presence of flavonoid compounds, which presents a mechanism of action associated with their adsorption of cell membranes, interaction with enzymes, or through the inhibition of nucleic acid synthesis and energy metabolism [33]. Results of CNps showed MIC values of 15-25 μg/mL, presenting better results against Gram-positive bacteria (*Staphylococcus aureus*). The antimicrobial activities of the chitosan against Gram-positive bacteria have been associated with its ability to bind non-covalently with teichoic acids incorporated in the peptidoglycan layer of the bacteria [34]. While, the activity against Gram-negative bacteria is correlated to the chelation effect of chitosan with different (Ca^2+^, Mg^2+^, etc.) and the electrostatic interaction of chitosan and the anionic parts of the lipopolysaccharide at the outer membrane [34]. 

The results of PL-CNps showed a reduction in the MIC value (10.31 μg/mL) compare to the free extract. The modification of CNPs with PLW phenolic extract involved the amine group of chitosan, resulting in a Z potential reduction after encapsulation (−3.8 ± 0.28 mV). However, phenolic compounds in the PLW extract also presented antibacterial properties, which can compensate for the ‘‘lost’’ of antibacterial properties of the amine group on CNPs [30]. To determine synergic effects, MIC values were converted into inhibition %. The results indicated that a synergic effect was observed in *Escherichia coli* and *Salmonella typhimurium*, due to the Ee value (4.72 + 4.0 - [(4.72 × 4.0)/100] = 8.53 %) was lower than the experimental value of inhibition by antibacterial tests (9.69%) [31]. Lee et al. [35] also observed an increase in the antibacterial activity of chitosan after conjugation with hydroxycinnamic acids, exhibiting activity against *Bacillus subtilis* (MIC: 2 μg/mL), *Enterococcus faecalis* (MIC: 16 μg/mL), and *Listeria monocytogenes* (MIC: 32 μg/mL). Antimicrobial activity of PL-CNps can be associated with their size, allowing them to exhibit a better activity due to a greater surface area [36]. In contrast, no synergism [31] was observed in PL-CNps against *Staphylococcus aureus* bacteria since the calculated values of expected additive response (Ee: 3.54 + 6.66 - [(3.54 × 6.66)/100] = 9.97 %) was higher than the observed percent of inhibition on *Staphylococcus aureus* (9.69%).

## 3. Materials and Methods

### 3.1. Plant Material and Microorganisms

Peels, pulps, and seeds from the industrial process of Persian Lemon (*Citrus latifolia)* waste (PLW) were collected during the month of January 2016 from a local market in Oxkutzcab, Yucatán, Mexico. The PLW was dried using a steam dehydrator (148-09, Jersa, Mexico) with air circulation at 60 °C for 48 h and then the dried material was finely milled (200 grinder, Pulvex Mexico), sieved (metal sieve <0.500 mm) and stored in dark plastic bags until they were used [37]. 

The strains of microorganisms used were *Escherichia coli* ATCC 25922, *Staphylococcus aureus* ATCC 25923 and *Salmonella typhimurium* ATCC 14028. All strains were provided by Centro de Investigación y Asistencia en Tecnología y Diseño del Estado de Jalisco Culture Collection (Yucatan, Mexico).

### 3.2. Chemicals and Reagents

Low molecular weight chitosan sample (average molecular weight <300 kDa; a degree of deacetylation = 83%) was produced from shrimp shells (*Litopenaeus vannamei*) using a UAE. Analytical reagents and standards used were: Folin–Ciocalteu reagent (2N), sodium carbonate (Na_2_CO_3_), 1,1-diphenyl-2-picrylhydrazyl (DPPH), 2,2′-azino-bis-3-ethylbenzothiazoline-6-sulphonic acid (ABTS), 6-hydroxy-2,5,7,8-tetramethylchroman-2-carboxylic acid (Trolox), potassium persulfate (K_2_S_2_O_8_), *ρ*-iodonitrotetrazolium, Mueller-Hinton broth, sodium tripolyphosphate (TPP), gallic acid, chlorogenic acid, caffeic acid, eriocitrin, ellagic acid, *ρ*-coumaric acid, sinapic acid, diosmin, hesperidin, catechin and quercetin, HPLC grade methanol, acetic acid, and acetonitrile were purchased from Sigma-Aldrich (Toluca, State of Mexico, Mexico). Ultra-pure water was prepared in a Milli-Q water filtration system (Millipore, Bedford, MA, USA). 

### 3.3. Optimization of UAE

For UAE, a 130W Ultrasonic Liquid Processor (GEX130PB, Sonics and Materials Inc., Newtown, USA) with a 13 mm diameter probe at a frequency of 20 kHz was used to extract the phenolic compounds from PLW. Briefly, 1 g of PLW powder was immersed in 40–50 mL of each concentration of the solvent, according to the conditions evaluated. The samples were then sonicated and a cold water bath was used to maintain the temperature at 50 ± 0.5 °C. After applying the ultrasound, the processed samples were filtered through a Whatman No. 4 filter paper using a Buchner funnel which was connected to a vacuum pump. Finally, extracts were collected into amber vials and stored in a refrigerator at 4 °C until the analysis. Conventional extraction was performed by maceration of PLW powder (1 g) with 40 mL of 50% aqueous ethanol. The samples were then homogenized using magnetic stirred for 1 h at room temperature (25 °C). After extraction, the extracts were filtered and collected into amber vials as described above.

#### 3.3.1. Response Surface Methodology 

A Response Surface Methodology (RSM) was used to optimize the extraction parameters of TPC recovery expressed as mg GAE/g dw in PLW. A three-level four-factorial Box–Behnken design was used for UAE with ethanol. The three levels of each of the four variables were coded and 27 runs were analyzed in random order. The four variables for UAE were ethanol concentration (%, X_1_), extraction time (min, X_2_), amplitude (%, X3), and solid to liquid ratio (g/mL). The three levels were X1 (50, 60 and 70%), X2 (10, 12.5 and 15 min), X3 (60, 75 and 90 %), and X4 (1 g/40, 1 g/45 and 1 g/50 mL). 

The TPC recovery data were fitted to a second-order polynomial equation (Equation (2)):

(2) $$Y=B_{0}+\overset{k}{\underset{i=1}{\sum }}BiXi+\overset{k}{\underset{i=1}{\sum }}BiiX^{2\;}+\overset{k}{\underset{i>j}{\sum }}BijXj^{\;}+E$$

where, Y represents the response variable; B_0_ corresponds to a constant coefficient; Bi, Bii, and Bij are the regression coefficients of the linear, quadratic and interactive terms, respectively; and Xi and Xj represent the independent variables. The regression coefficients of individual linear, quadratic, and interaction terms were determined using analysis of variance (ANOVA) according to the Fisher test (F-Ratio) and probability p (P-value). To indicate the significance of the terms, p-values from ANOVA was used for the response variable using the full model. To verify the suitability of the model, an additional extraction was carried out according to the optimal predicted conditions to compare the experimental data with the values predicted. To visualize the relationship between the response variable and experimental levels and to predict the optimum conditions, the regression coefficients were used to generate 3-D surface plots from the fitted polynomial equation. 

### 3.4. TPC Determination

TPC was determined with Folin-Ciocalteau reagent with some modifications [37]. The absorbance of samples was measured using a UV-visible spectrophotometer (Biomate 3S, Thermo Fisher Scientific, Inc., Wathan, MA, USA) at 765 nm. TPC analysis was performed in triplicate and values were expressed as mg of gallic acid equivalents (GAE) per g of dry weight (dw) samples through a calibration curve of gallic acid (R^2^ = 0.995). 

### 3.5. Phenolic Compounds Identification and Quantification by UPLC-PDA Analysis 

Previous to the UPLC-PDA analysis, the extracts obtained by optimal UAE conditions were concentrated using a rotavapor (R-215, Buchi, Switzerland) at 50 °C, 100 rpm, and 220 mbar. The chromatographic determinations were performed using a Waters UPLC Acquity H Class (Milford, MA, USA) equipped with a quaternary pump (UPQSM), autosampler injector (UPPDALTC) and PDA λ photodiode array detector (UPPDALTC). Empower 3 software (Waters, 2010, Milford, MA, USA) was used for data acquisition and processing. Chromatographic separation of the phenolic compounds was carried out according to reference [37]. Quantification was performed using analytical standard curves prepared individually and by mixing analytical grade standards (gallic acid, chlorogenic acid, caffeic acid, eriocitrin, ellagic acid, *ρ*-coumaric acid, sinapic acid, diosmin, hesperidin, catechin, and quercetin) at concentrations from 1 to 50 ppm. 

### 3.6. Encapsulation of Phenolic Compounds by Ionic Gelation

The CNps were prepared through the ionic gelation technique reported by Medina-Torres et al. [38]. A solution of 0.2% chitosan was prepared and the pH was adjusted to 4.5. The sample was mixed with a 0.1% TPP solution at a ratio of 3:1 chitosan:TPP (*v/v*). The mixture was stirred for 1 h at room temperature. Then, the mixture was sonicated using a 130W Ultrasonic Liquid Processor (GEX130PB, Sonics and Materials Inc., Newtown, USA) at a wave amplitude of 50% radiation during 8 min to obtain the CNps. For phenolic encapsulation, the extracts obtained at optimum UAE conditions and the solution containing CNps were mixed at a 1:1 ratio (*v/v*). The mixture was stirred and sonicated as described for CNps formation. Finally, the solution was centrifuged (Spectrafuge 24D, Labnet, International Inc., USA) at 14000 g for 15 min at 25 °C. The supernatant was separated from the pellet where the PL-CNps were presented [10].

Encapsulation efficiency (EE) was determined by the quantification of residues of phenolic compounds presents in the supernatant after PL-CNps formation and expressed in percentage (%) according to (Equation (3)):

(3) $$\%EE:\;\frac{C_{i}-C_{s}}{C_{i}}\;\times \;100\;\;\;\;\;\;\;\;\;\;\;\;\;\;\;\;\;\;\;\;$$

where, Ci is the phenolic compounds concentration used for the developed of PL-CNps and Cs phenolic compounds concentration in the supernatant. The phenolic compounds were determined through UPLC-PDA analysis as described above.

### 3.7. PL-CNps Characterization 

#### 3.7.1. Size Particle, Z Potential, and Polydispersity Index (PDI) of PL-CNps

Size particle, Z potential, and polydispersity index (PDI) were measured using an automated capillary electrophoresis device (Zetasizer Nano ZS90, Malvern Instruments Ltd., Worcestershire, UK). The samples were diluted to a concentration of approximately 0.01% (*w*/*v*) using deionized water and then the index of refraction was determined using a digital refractometer (NAR-2T Atago, USA) before analysis. Diluted samples were performed at pH ranging from 4 to 5. The Z potential was determined by measuring the direction and velocity of the dispersions as they moved along the applied electric field. The software of the equipment converted the electrophoretic mobility measuring into Z potential values using the Smoluchowsky model. On the other hand, the size distributions of samples were determined according to the Stokes-Einstein equation (Equation (4)):

Rh = kBT/6Sd(4)

where, Rh is z-average radius, KB is the Boltzmann constant, T is the absolute temperature, S is the dynamic viscosity of the solvent, and d is the z-average translational diffusion coefficient [39]. 

#### 3.7.2. Fourier Transformed-Infrared (FT-IR) Analysis 

The infrared spectra were recorded with an FTIR spectrophotometer (Cary 630, Agilent Technologies, USA), equipped with a diamond crystal. Dried samples were brought into contact with the diamond crystal using a geometry adjustment of 60 units of force gauge, and the absorbance at wavelengths from 600 cm^−1^ to 4000 cm^−1^ was measured. FT-IR estimated the absorption frequency of light due to excitation in the energy level of the substance in the infrared region.

### 3.8. PL-CNps Biological Activity

Analysis of biological activity was performed by determination of antioxidant and antibacterial evaluation of PL-CNps. Antioxidant activity was determined through two different methods, one based on the scavenging of DPPH and the other by the radical inhibition test of ABTS. The DPPH and ABTS tests were carried out in line with the method used by Covarrubias-Cárdenas et al. [37]. Results were expressed as μmol of Trolox Equivalent (TE)/g dw, according to the calibration curve (R^2^ = 0.992 for DPPH and R^2^ =0.990 for ABTS). The percentage of radical inhibition of DPPH and ABTS was calculated according to Equation (5):

(5) $$\mathrm{DPPH}\;\mathrm{or}\;\mathrm{ABTS}\;\mathrm{Inhibition}\;\left(\%\right)=\;\frac{A\;control-A\;sample\;}{A\;control}\;\times \;100\;\;\;\;\;\;\;\;\;\;\;$$

where A control is the absorbance of the control, and A sample is the absorbance of the sample.

Antibacterial activity was performed by Minimum Inhibitory Concentration (MIC) determination based on the methodology reported by Plascencia-Jatomea et al. [31] with some modifications. The serial dilution method was used for determination of MIC. The samples (50 µL) was distributed in a sterile nonpyrogenic 96 well cell culture plate (Costar^®^ Assay Plate, Corning Inc., Corning, NY, USA). A mixture of 100 µL of Mueller-Hinton medium and 50 µL of bacterial inoculum was also added to the culture plate. The MIC value was defined as the lowest concentration that demonstrated no visible growth after incubation at 37 °C during 24 h. The *ρ*-iodonitrotetrazolium solution was used as a color indicator, where purple color indicated visible microbial growing. NaCl solution (0.09%) was used as a negative control. The percentage of bacterial growth inhibition (*Escherichia coli*, *Staphylococcus aureus,* and *Salmonella typhimurium*) was calculated by Equation (6):

(6) $$\mathrm{Bacterial}\;\mathrm{growth}\;\mathrm{inhibition}\;\left(\%\right)=\;\frac{1\frac{\mu g}{ml}of\;sample\;}{MIC\;value\;of\;sample\left(\frac{\mu g}{ml}\right)}\;\times \;100.$$

The biological activity of the combination of phenolic extract and CNps and synergism effect were determined using Limpel’s formula by Equation (7)

Ee = X + Y − (XY/100)(7)

where Ee was the expected additive responses for the phenolic extract and CNps, such as X and Y were the percentages of inhibition of the phenolic extract or CNps, according to antioxidant or antibacterial test. Thus, if the combination of the two agents produced any value of inhibition greater than Ee, then synergism existed [31].

### 3.9. Statistical Analysis 

All experiments were conducted in triplicate and data were reported as the mean ± SD. The results obtained were subjected to Multifactorial Analysis of Variance (ANOVA) and LDS means comparison analysis. Significance was established at p using a *p* ≤ 0.05. The data from Box–Behnken experimental design were analyzed by least square multiple regression methodology to fit the polynomial models in UAE optimization. Data analysis and response surfaces were conducted using the Statgraphics Centurion XVI.I software (Statistical Graphics Corp., Manugistics, Inc., Cambridge, MA, USA).

## 4. Conclusions

Persian lemon waste has a large potential as a raw material for recovery of phenolic compounds by UAE for PL-CNps development. The influence and optimal conditions of ethanol concentration, extraction time, amplitude, and the solid-liquid ratio on TPC were proven using RSM analysis. The CNps could be used as an excellent alternative for the encapsulation of phenolic compounds from PLW, reporting EE up to 80%. Additionally, PL-CNps presented an improvement on its antioxidant and antibacterial activity, which suggests a synergistic effect between phenolic compounds from PLW and CNps. Such results indicate that PLW presents a biological potential for the production of value-added compounds. 

## Figures and Tables

**Table 1:** Experimental and predicted values of Total Phenolic Content (TPC) obtained by ultrasound-assisted extraction (UAE) with different conditions used in the randomized Box–Behnken design.

Experimental Run	Independent Variables				TPC (mg GAE/g dw)	
	X_1_ (%)	X_2_ (min)	X_3_ (%)	X_4_ (g/mL)	Experimental	Predicted
1	70	12.5	80	1/40	12.40 ± 0.23	13.39
2	60	15.0	60	1/45	11.62 ± 0.44	12.02
3	60	15.0	100	1/45	11.07 ± 0.29	10.52
4	60	12.5	60	1/40	10.53 ± 0.20	9.70
5	70	12.5	60	1/45	10.32 ± 0.14	9.71
6	60	12.5	100	1/40	12.04 ± 0.51	12.04
7	60	12.5	100	1/50	14.18 ± 0.39	14.75
8	60	10.0	80	1/40	12.22 ± 0.23	12.68
9	50	12.5	100	1/45	12.35 ± 0.01	12.96
10	70	12.5	80	1/50	14.77 ± 0.58	14.50
11	60	10.0	60	1/45	8.10 ± 0.01	8.78
12	60	15.0	80	1/40	12.86 ± 0.33	12.08
13	50	10.0	80	1/45	14.55 ± 0.44	14.49
14	60	12.5	60	1/50	12.74 ± 0.49	12.41
15	70	12.5	100	1/45	13.87 ± 0.01	13.83
16	60	12.5	80	1/45	14.49 ± 0.32	14.40
17	70	10.0	80	1/45	13.54 ± 0.29	13.59
18	60	12.5	80	1/45	14.86 ± 0.39	14.40
19	60	10.0	100	1/45	15.71 ± 0.02	14.96
20	60	10.0	80	1/50	15.35 ± 0.29	15.40
21	70	15.0	80	1/45	12.47 ± 0.17	12.99
22	60	12.5	80	1/45	14.35 ± 0.08	14.40
23	50	15.0	80	1/45	13.50 ± 0.40	13.89
24	60	15.0	80	1/50	15.37 ± 0.06	14.80
25	50	12.5	60	1/45	11.81 ± 0.20	12.40
26	50	12.5	80	1/50	17.84 ± 0.01	17.01
27	50	12.5	80	1/40	12.50 ± 0.59	12.69

**Table 2:** Phenolic compounds identification and quantification by ultra-performance liquid chromatography coupled photo diode array (UPLC-PDA) analysis from waste Persian Lemon obtained by UAE and maceration methods.

Method	Phenolic Compounds Profile and Content (mg/g dw)								TPC (mg/g dw)
	Cat	Eri	Cu. Ac	Sin. ac	Dio	Hes	Neo	Nar	
**MCT**	0.20 ± 0.0 ^b^	5.12 ± 0.1 ^b^	0.63 ± 0.0 ^b^	0.71 ± 0.0 ^b^	2.00 ± 0.0 ^b^	2.01 ± 0.0 ^b^	0.27 ± 0.0 ^b^	0.25 ± 0.0 ^b^	11.19 ± 0.1 ^b^
UAE	2.92 ± 0.1 ^a^	20.71 ± 0.1 ^a^	2.86 ± 0.0 ^a^	3.67 ± 0.1 ^a^	18.59 ± 0.1 ^a^	7.30 ± 0.1 ^b^	1.63 ± 0.0 ^a^	0.44 ± 0.0 ^a^	58.13 ± 0.4 ^a^

MCT: Maceration, Chat:Catechin, Eri:eriocitrin, Cu. Ac:*p*-coumaric acid, Sin. Ac:sinapic acid, Dio:diosmin, Hes:hesperidin, Neo:neohesperidin, Nar:naringin. Values are the mean ± SD of 3 independent replicates. Different letters in the same column indicate that mean values are statistically different (*p* < 0.05).

**Table 3:** Biological activity of phenol-loaded chitosan nanoparticles (PL-CNps), free PLW extract and chitosan nanoparticles (CNps).

Treatment	Antioxidant Activity (μmol TE/g dw)				Antibacterial Activity MIC (μg/mL)					
	ABTS		DPPH		*E. coli*		*S. typhimurium*		*S. aureus*	
	Trolox eq.	I%	Trolox eq.	I%	MIC	I%	MIC	I%	MIC	I%
**Free PLW**	549.08 ± 14.0 ^b^	57.4	592.55 ± 3.9 ^b^	58.8	21.17 ± 0.0 ^b^	4.72	21.17 ± 0.0 ^b^	4.72	28.22 ± 0.0 ^a^	3.54
**CNps**	36.39 ± 5.7 ^c^	9.7	9.0 ± 0.0 ^c^	2.2	25.00 ± 0.0 ^a^	4.00	25.00 ± 0.0 ^a^	4.00	15.00 ± 0.0 ^b^	6.66
**PL-CNps**	795.37 ± 1.9 ^a^	80.4	687.81 ± 14.3 ^a^	66.0	10.31 ± 0.0 ^c^	9.69	10.31 ± 0.0 ^c^	9.69	10.31 ± 0.0 ^c^	9.69

I%: percentage of inhibition. Values are the mean ± SD of 3 independent replicates expressed as the inhibition percentage and μm Trolox/g dw for antioxidant activity and as MIC (μg/mL) and inhibition percentage for antibacterial activity. Different letters in the same column indicate that mean values are statistically different (*p* < 0.05).
